# Nutritional intakes in children with Prader–Willi syndrome and non-congenital obesity

**DOI:** 10.3402/fnr.v59.29427

**Published:** 2015-12-08

**Authors:** Daniela A. Rubin, Jill Nowak, Erin McLaren, Monzeratt Patiño, Diobel M. Castner, Marilyn C. Dumont-Driscoll

**Affiliations:** 1 Department of Kinesiology, California State University, Fullerton, CA, USA; 2 Department of Endocrinology, Children's Hospital of Orange County, Orange, CA, USA; 3 Department of Pediatrics, College of Medicine, University of Florida, Gainesville, FL, USA

**Keywords:** macronutrients, micronutrients, nutritional deficiencies, childhood obesity

## Abstract

**Background:**

Individuals with Prader–Willi syndrome (PWS) have extremely regulated diets to prevent the development of morbid obesity.

**Objective:**

This study evaluated potential deficiencies in macro and micronutrients in a cohort of youth with PWS and compared them to a group of children with non-congenital obesity and to US national recommendations.

**Design:**

Participants were 32 youth with PWS (age=10.8±2.6 years, body fat=46.7±10.1%) and 48 children without PWS but classified as obese (age=9.7±1.2 years, body fat=43.4±5.7%). Participants’ parents completed a training session on food recording before completing a 3-day food record during a typical week including a weekend day and two weekdays, as well as a screening form indicating nutritional supplements use.

**Results:**

Youth with PWS reported less calories (1,312±75 vs. 1,531±61 kcal, *p=*0.03), carbohydrate (175±10 vs. 203±8 g), and sugars (67±5 vs. 81±4 g; *p=*0.04 for both) than obese. Youth with PWS consumed more vegetables (1.1±0.1 vs. 0.6±0.1 cups) and more of them met the daily recommendation (*p<*0.01 for both). Likewise, youth with PWS consumed more calcium than obese (899±53 vs. 752±43 mg) and more of them met the recommended daily dose (*p=*0.04 for both). The majority of participants in this study did not meet the vitamin D recommendation.

**Conclusion:**

Despite consuming less calories, youth with PWS had a similar proportion of macronutrients in their diet as children with obesity. Micronutrient deficiencies in calcium and vitamin D in youth with PWS were noted despite a third of youth with PWS consuming multivitamin supplements. Special attention must be paid to the diets of youth with PWS and with obesity to ensure they are meeting micronutrient needs during this period of growth and development.

Prader–Willi syndrome (PWS) is a neurobehavioral disorder resulting from an alteration in the expression of the paternal chromosome 15 at the locus q13–q15. Its prevalence ranges from 1 in 10,000 to 1 in 20,000 live births (1). The syndrome's hallmark is neonatal hypotonia often accompanied by failure to thrive with the subsequent development of hyperphagia and an insatiable appetite that can lead to morbid obesity. However, people with PWS also manifest other clinical features such as cognitive disability, rigid thinking, behavioral challenges, growth hormone deficiency, poor muscle mass and tone, and low stamina.

Five distinct nutritional phases have been delineated for individuals with PWS, with the first phase taking place *in utero* (2). The second phase comprises two sub-phases in which no signs of hunger are present from birth to 9 months followed by the development of a normal appetite developing between 9 and 25 months. The third phase is subdivided into two sub-phases in which there is weight gain but no increased food consumption, and then followed by continued weight gain with the development of an interest in food beginning at about 4.5–8 years of age. Phase 4 is defined by the onset of hyperphagia at about 8 years of age. The fifth and last phase takes place in adulthood with the appetite no longer insatiable. Not all people with the syndrome go into this phase at the same age.

Individuals with PWS have a lower caloric need by about 20–40% less than people without the syndrome (3, 4). Recommendations for caloric intake in PWS for ensuring weight maintenance have evolved from 8.4–14.6 kcal/cm ht (5) to 10.0–14.0 kcal/cm ht (6). For weight reduction, the recommended range is 7–9 kcal/cm ht (5, 6).

Weight management is difficult in children with PWS because of lower caloric needs due to the lower lean muscle mass and lower spontaneous physical activity compared to controls (4, 7, 8). Two interventions in the United States tested different guidelines to achieve weight loss in people with PWS. Bonfig et al. (9) asked five adolescents (two female and three male) with PWS to consume 10 kcal/cm per day, resulting in a significant body mass index (BMI) reduction of 41.3–33 kg/m^2^ over 2 years. Miller et al. (10) evaluated changes in body composition on 63 youth, aged 2–15 years, while consuming 30–60 kcal/kg per day matched to their resting energy expenditure. Thirty-three of the 63 participants met both the recommended caloric intake and prescribed diet consisting of 30% fat, 45% carbohydrates, and 25% protein, with at least 20 g of fiber per day. Overall, the participants that complied with the specified diet achieved a larger decrease in body fat percentage than those only achieving the caloric recommendation (10). Moreover, Schmidt et al. (11) also showed maintained weight when children with PWS were on strict fat diet with 10 kcals/cm height per day.

Data on nutritional intake in people with PWS is scant, though there are three studies that describe macronutrient intake, frequency of consumption of fruits and vegetables, and micronutrient deficiencies. Lindmark et al. (12) demonstrated that seven young children (aged 3–4 years) who lived with their parents consumed less sugar, more protein, and less fat than Norwegian norms, but without the use of nutritional supplements, deficiencies were identified in Vitamin D, tocopherol, calcium, and iron. In the United States, Miller et al. (10) studying children with PWS aged 3–15 years, identified macronutrient intake as ranging between 10–23% for fat, 50–70% for carbohydrate, and 10–20% for protein with about 12.0 g or less of fiber per day. Nordstrom et al. (13) in Norway, assessed adults with PWS living in a supervised community setting and reported good daily consumption of fruits and vegetables with low consumption of juices and soft drinks, but poor intake of fish.

The American Heart Association and the United States Department of Agriculture (USDA) recommend about 1,200–1,400 calories a day for 4- to 8-year-old girls and boys, and about 1,800–2,200 calories a day for 14- to 18-year-old girls and boys (14, 15). Daily carbohydrate recommendations should constitute 45–65% of total calories, 10–30% of total calories for protein, and total fat intake limited to 25–35% (14, 15). Daily food recommendations include 1.5–2.0 cups of fruit along with 1.0–3.0 cups of vegetables for ages 4–8 and 14–18 years, respectively (14). For ages 1–8 years, children need 2.0–2.5 cups of dairy with ages 9–18 years needing 3.0 cups. Finally, 5.0 oz of grains a day for 4- to 8-year-old children to 8.0 oz a day for 14–18 year olds are recommended (15). For meats and beans, recommendations range from 3.0–4.0 oz per day for 4- to 8-year-old children to 5.5–6.5 oz per day for 14–18 year olds (15). Some nutrients that could be under-consumed include vitamin D, calcium, iron, potassium, and fiber (16).

Because of the restricted energy consumption to which youth with PWS must adhere to maintain a healthy weight, it is possible that deficiencies are present at the macro and the micronutrient level (10, 12, 13). Although youth who are obese but without PWS may be ingesting more calories than they need, they still may present nutritional intake deficiencies.

The purpose of this study was to determine the nutrient intake of children and adolescents with PWS, compare it with obese controls, and to the nutritional recommendations guidelines for Americans in 2015 (15, 16). It is the aim of this study to identify potential common deficiencies in those with and without PWS at the macro and micronutrient levels.

## Methods

### Participants

Participants were part of a larger study in which children completed a 24-week, home-based physical activity intervention (17). Data included in this study were obtained at baseline, prior to beginning the physical activity program. Youth with PWS were recruited via the Prader–Willi California Foundation, CHOC Children's Hospital (Children's Hospital of Orange County), the University of Florida Health Shands Children's Hospital, Prader–Willi syndrome Association (USA) and the study website (www.pws.fullerton.edu). Children without PWS were recruited through flyers, newspaper advertisements, the study website, word of mouth, as well as pediatricians’ referrals (University of Florida).

Data from 32 youth with PWS, aged 8–16 years, and 48 children, aged 8–11 years with non-congenital obesity (body fat percentage ≥95th percentile for age and sex, obese) (18), were analyzed. The PWS diagnosis (and sub-type when possible) was confirmed by medical records documenting genetic testing: deletion (*n*=14), uniparental disomy (*n*=7), either uniparental disomy or imprinting defect (*n*=3), and confirmed DNA methylation (*n*=8).

Youth with PWS also reported currently (*n*=24), previously (*n*=5), or never (*n*=3) using growth hormone replacement therapy. Other medications and supplements reported in youth with PWS were testosterone (*n*=2), CoQ10 (*n*=9), diabetes medications (*n*=7), albuterol (*n*=9), inhaled steroids (*n*=5), other asthma medications (*n*=5), and various other medications for allergies (*n*=3), behavior disorders (i.e. ADHD, depression, schizophrenia, bipolar disorder, anxiety) (*n*=5), skin issues (*n*=3), seizures (*n*=3), hypothyroidism (*n*=2), gastrointestinal/digestive disorders (i.e. reflux, constipation) (*n*=5) and sleep apnea (*n*=1). Children with non-congenital obesity reported taking inhaled steroids (*n*=4), other asthma medications (*n*=7), allergy medications (*n*=7), and medications for ADHD (*n*=2). This study was approved by the Institutional Review Boards from California State University, Fullerton, University of Florida Health Science Center, and the United States Army Medical Research and Materiel Command.

### Measurements and instruments

Upon arrival to the laboratory, written informed assent and consent were obtained from all participants and their parents. A medical history questionnaire was completed by the parent of the child participant. Children were measured for anthropometrics (stature to the nearest centimeter and body mass to the nearest kilogram) following standard procedures (19) and body composition was obtained following the manufacturer's protocol through a dual-energy x-ray absorptiometry scan (GE Healthcare, GE Lunar Corp., Madison, WI).

Parents of child participants then completed a nutrition training led by a registered dietitian. The training followed the parent completion of a nutrition screening form, and consisted of an instructional session, followed by a food portion quiz at the end of the training. During the instructional session, parents were taught types of food and beverage measurements, how to measure portion sizes, and how to properly complete a food record.

Parents were instructed to log in real time at home onto a food record their child's food and drink intake during two weekdays and one weekend day. The food record directed the parent to list all food and drinks with their brand, type, preparation, amount offered, amount consumed, and location of food consumption. Parents also were provided a set of measurement cups and spoons to take home. Completed food records were then mailed back to the research staff in prepaid envelopes provided to the parent.

Parents were also asked to complete a nutrition screening form, which inquired about previous nutritional counseling from a registered dietician, placement on a specific or calorie-limited diet, and the use of nutritional supplements or multivitamins.

### Data screening and analysis

Upon receipt, the food records were screened to ensure that the parent recorded at least two weekdays and one weekend day. Data from the food records were entered into the Food Processor program, version 10.12.0 (ESHA Research, Salem, OR). Weekly averages were calculated in the Food Processor program and later inputted into IBM SPSS Statistics 20 for Windows (SPSS, Inc., Chicago, IL) for further analyses. Independent samples *t*-tests were used to determine possible group differences for participant characteristics. Frequencies of use of nutritional supplements were included. Group differences for total kilocalories and percentage of intake for macronutrients were evaluated using analyses of covariance, controlling for age to account for the larger age range of youth with PWS. Group differences for frequency of meeting micronutrient recommendations for calcium, vitamin D, iron, and B vitamins, as well as intake of fruits, vegetables, grains, protein, and dairy were evaluated using Chi-square analyses. Significance level was set at *p*<0.05 for all analyses.

## Results

### Participant characteristics

Ethnic breakdown distribution for youth with PWS included Asian (*n*=2), African–American (*n*=1), Hispanic (*n*=7), Caucasian (*n*=21), and other (*n*=1). For children without PWS, the ethnic breakdown included Asian (*n*=4), Hispanic (*n*=29), Caucasian (*n*=11), and other (*n*=4). Participant characteristics are presented in Table 1. As expected, PWS were significantly older than the controls due to the wider age range, though the controls had significantly greater BMI *z*-score than the youth with PWS. There was a trend toward the PWS group presenting a higher body fat percentage than controls. All other characteristics were similar between groups (*p*>0.05).

**Table 1:** Participant characteristics by group (PWS vs. obese), presented as frequencies or mean±standard deviation

	PWS (*n*=32)	Obese (*n*=48)	*p*
Sex (M/F)	18/14	29/19	
Age (y)	10.8±2.6*	9.7±1.2	0.014
Stature (cm)	145.0±13.4	144.6±8.1	0.873
Body mass (kg)	59.78±25.84	56.13±11.46	0.392
BMI *z*-score	1.73±0.92*	2.05±0.41	0.035
Body fat (%)	46.7±10.1	43.4±5.7	0.068
Lean mass (kg)	28.67±10.09	30.01±4.96	0.435

BMI, body mass index

* *p*<0.05.

### Dietary patterns and nutritional supplement use

Ninety-eight completed food records (PWS: *n*=40; obese: *n*=58) were received from participants (17). Food records from 32 youth with PWS and 48 children without PWS were deemed valid for use in this study (18.4% of food records were omitted). Records were valid if they included data on two weekdays and one weekend day.

Data from the nutrition screening form were obtained from 77 participants. Dietary counseling, from a registered dietitian, was reported by 83.9% of the PWS group (*n*=31) and 17.4% of the obese group (*n*=46), with 80.6% of PWS (*n*=25) and 6.7% of obese (*n*=3) following a special diet or calorie limit. One parent of an obese child declined answering the previous question. Supplement use was reported in the PWS group for consuming multivitamins (*n*=13), vitamin C (*n*=1), vitamin D (*n*=6), calcium (*n*=4), and fish oil (*n*=3), while children without PWS reported consuming the same or less supplements: multivitamins (*n*=8), vitamin C (*n*=1), and vitamin D (*n*=1).

### Nutritional dietary intake

Daily total caloric intake and macronutrients consumption are presented in Table 2. Youth with PWS consumed less total daily kilocalories and grams of total carbohydrates and sugars than obese children without PWS. Additionally, there was a trend toward statistical significance for youth with PWS consuming less grams of fat compared to obese controls. There were no statistical differences in the grams of protein consumed between the groups. When each of the macronutrient consumptions was presented as a percentage of the total kilocalories consumed, both groups consumed similar proportions for all macronutrients.

**Table 2:** Daily caloric intake and macronutrients consumption, controlling for age, by group, presented as mean±standard error

	PWS	Obese	*p*
Total intake (kcal)	1312.2±74.7*	1530.7±60.6	0.029
Fat			
Intake (g)	43.7±3.3	51.4±2.7	0.081
% of total kcal	28.9±1.0	29.8±0.8	0.493
Protein			
Intake (g)	61.7±3.8	67.7±3.1	0.231
% of total kcal	18.9±0.7	17.9±0.6	0.298
Carbohydrates			
Total Intake (g)	174.5±10.0*	203.4±8.1	0.036
% of total kcal	54.0±1.0	53.3±0.8	0.626
Sugars (g)	66.8±4.9*	80.5±4.0	0.035

* *p*<0.05.

In terms of food groups’ consumption (see Table 3), analyses showed that youth with PWS consumed a greater amount of vegetables (cup) than obese controls. Additionally, a larger proportion of youth with PWS than without met the daily intake recommendation for vegetables. There were no other group differences for food groups’ consumption.

**Table 3:** Daily food group consumption by group, controlling for age, presented as mean±standard error, and frequency of children who met the recommendation

	Intake			Met recommendation (yes/no)		
		
	PWS	Obese	*p*	PWS	Obese	Chi-square *p*
Fruit (cup)	1.0±0.1	1.2±0.1	0.210	11/21	23/25	0.257
Vegetable (cup)	1.1±0.1*	0.6±0.1	0.001	10/22	3/45	0.005
Grain (oz)	4.2±0.3	4.5±0.3	0.448	13/19	26/22	0.261
Protein (oz)	4.2±0.5	4.4±0.4	0.690	17/15	29/19	0.645
Dairy (cup)	1.6±0.1	1.4±0.1	0.143	6/26	4/44	0.301

* *p*<0.05; recommendation determinations based on unadjusted values.

Data regarding micronutrient consumption is presented in Table 4. Youth with PWS consumed a greater amount of calcium (mg) than obese controls. Also, a larger proportion of youth with PWS than without were more likely to meet calcium daily intake recommendation. Of note, Vitamin B_12_ data from two youths with PWS were deemed outliers and were omitted from analyses. Both groups consumed similar amounts for all other micronutrients.

**Table 4:** Daily micronutrient consumption by group, controlling for age, presented as mean±standard error, and frequency of children who met the recommendation

	Intake			Met recommendation (yes/no)		
		
	PWS	Obese	*p*	PWS	Obese	Chi-square *p*
Calcium (mg)	898.5±53.4*	751.6±43.2	0.039	5/27	1/47	0.035
Vitamin D (µg)	6.9±0.8	5.5±0.6	0.155	2/30	0/48	0.157
Iron (mg)	11.7±0.9	12.8±0.8	0.383	32/0	46/2	0.514
Vitamin B						
B1 (mg)	1.4±0.1	1.7±0.1	0.176	26/6	44/4	0.301
B2 (mg)	1.9±0.1	1.8±0.1	0.908	30/2	46/2	1.000
B3 (mg)	1.7±15.4	1.4±18.9	0.211	27/5	43/5	0.732
B6 (mg)	2.0±0.2	1.8±0.2	0.374	29/3	38/10	0.225
B12 (µg)	5.1±0.5	4.4±0.4	0.295	25/5	44/4	0.294

* *p*<0.05; recommendation determinations based on unadjusted values.

## Discussion

This study demonstrates similarities and differences in nutritional intake between youth with PWS and obese controls. Overall, youth with PWS consumed fewer calories and less grams of carbohydrate (and more specifically sugar) than the control group of children with obesity. Additionally, it was demonstrated that a considerable proportion of all children in this study did not meet the vegetable, fruit, and dairy consumption recommendations. Supplementation with a multivitamin was common in 40% of youth with PWS; thus, deficiencies in B vitamins were not observed in the majority of these children. However, key deficiencies were observed for calcium and vitamin D not only in PWS but also in the obese controls.

Youth with PWS consumed 14% fewer calories than the obese controls as about 80% of the parents of youth with PWS indicated that their child was on a restricted calorie diet. This difference in calories between PWS and obese controls is smaller than the 20–40% less calorie consumption that was expected based on caloric restriction recommendations for PWS (2, 3). The smaller calorie restriction in our cohort was possibly due to several reasons. All children who participated in this study were about to begin a home-based physical activity intervention (17) and perhaps the parents of children without PWS were already implementing measures to restrict calories. Also, although parents completed the food record for all youth with PWS, those without the syndrome completed the records jointly with their children and underreporting might have taken place (20); which is not uncommon in people with obesity.

Despite consuming about 14% less calories, those with PWS consumed comparable grams of protein. Both groups consumed more than 0.8 g/kg body mass of protein, adequately meeting the recommendations for lightly active children. Participants with PWS consumed fewer grams of carbohydrate and potentially less fat than those without PWS. This finding is in line with a previous report in young children with PWS whose parents reported a lower consumption of fats and sugar than children without the syndrome (10).

In terms of recommended consumption of food groups, both groups reported eating about 1.0 cup of fruit per day, with more than half of the children not meeting the recommendation. For vegetables, a larger proportion of youth with PWS than the obese controls met the recommendation of 2.0 cups a day. This may be due to those with PWS being on a low-calorie diet and more likely to consume more vegetables due to the caloric restriction and/or the received counseling by a registered dietician. In terms of those children without PWS, our results further emphasize the need for nutritional education in this group regarding increased vegetable and fruit consumption as has been shown before for children in the United States (16).

While not all children in either group met the grains and protein recommendations, more than half of children without PWS met the grain recommendation while more than half of all children met the protein intake recommendation. It is not surprising that the obese controls consumed more calories through carbohydrates (specifically mainly because of sugars), and fat compared to those with PWS. This could be related to caloric restriction measures and counseling received in those with PWS.

In terms of micronutrients, youth with PWS consumed greater quantities of calcium, with four of them reporting calcium supplementation. However, while only 6 of 32 youth with PWS met the dairy consumption, even fewer (4 of 48) obese children met it. Our findings support previously reported under-consumption of calcium in children (16) and the need to promote dairy consumption in this population.

Other studies have also demonstrated under-consumption of iron and vitamin D in children with and without PWS (10, 12, 16). In our study, the majority of children met the iron recommendations based on their age and sex. However, our results once again show that a very limited number of children met the vitamin D recommendations. Vitamin D can also be synthesized from sunrays exposure; as these participants resided in California and Florida, they likely had opportunities for adequate sun exposure. However, due to sunscreen application, restricted outdoor play in minority populations due to unsafe neighborhoods, and increased screen time, these children could have had limited sun exposure. Therefore, a deficiency in vitamin D intake is noteworthy.

We found no differences between the groups for the vitamin B complex with about half of these children meeting the recommendations. In the United States, data have not demonstrated deficiencies for these vitamins, which is probably due to adequate consumption of grains and protein (16). Therefore, though youth with PWS may be consuming less grain than recommended, the addition of the multivitamin compensates to prevent deficiencies in the intake of the vitamin B complex.

This study had some methodological limitations. The study only included a group of youth with PWS and a comparison group of non-syndromic obesity but no healthy weight controls. Therefore, generalizations can only be made to this particular group of children. Additionally, a large proportion of youth with PWS (83.9%) received nutritional counseling compared to those without PWS (17.4%). Thus, differences observed between PWS and controls in the food group consumption and calories may be related to the lack of nutritional counseling. However, this study was not designed to determine the origin of these differences.

## Conclusion and recommendation

This study demonstrates that youth with PWS aged 8–16 years consumed less calories than obese children aged 8–11 years without the syndrome. Despite lower caloric intake than the control group, the majority of these youth with PWS appeared to consume adequate amounts of protein and the recommended proportions of carbohydrate and fat for the calories consumed. However, a fair number of these youth with PWS still did not meet the recommendations for most food groups, with the exception of protein. In particular, a very small proportion of these youth with PWS met the daily recommendation for dairy products. More than a third of these youth also supplemented their diet with multivitamins (with or without calcium), and a few with calcium, to compensate for deficiencies in food intake. Considering the deficiencies noted for calcium and vitamin D, the use of nutritional supplements for these nutrients is highly recommended in PWS.

Parents of children with PWS face a daily challenge in managing their children's food intake given the hyperphagia and other neurobehavioral symptoms of this disorder. Overall, while the majority of children with PWS had received dietary counseling from a registered dietician prior to enrolling in this study, a number of nutritional deficits were identified and deserve additional directive counseling. Moreover, this study demonstrates the need of focusing attention and guidance on proper dietary intake in obese children without the syndrome, especially as it relates to increasing fruits, vegetables, and dairy consumption and caloric needs to prevent obesity.
